# Supplementary material for: Effects of Pharmaceutical Substances with Obesogenic Activity on Male Reproductive Health
Source: Int J Mol Sci. 2024 Feb 15;25(4):2324. doi: 10.3390/ijms25042324 (PMC10889417; doi:10.3390/ijms25042324)

Supplementary material

Illustration of different pharmacological agents with obesogenic activity classified according to the ATC/DD Index (https://www.whocc.no/atc_ddd_index/, accessed on 28 December 2023). Molecular structures retrieved from ChemSpider and PubChem.

Figure S1
A: Alimentary tract and metabolism

A10:Antidiabetics

A10A: Insulin

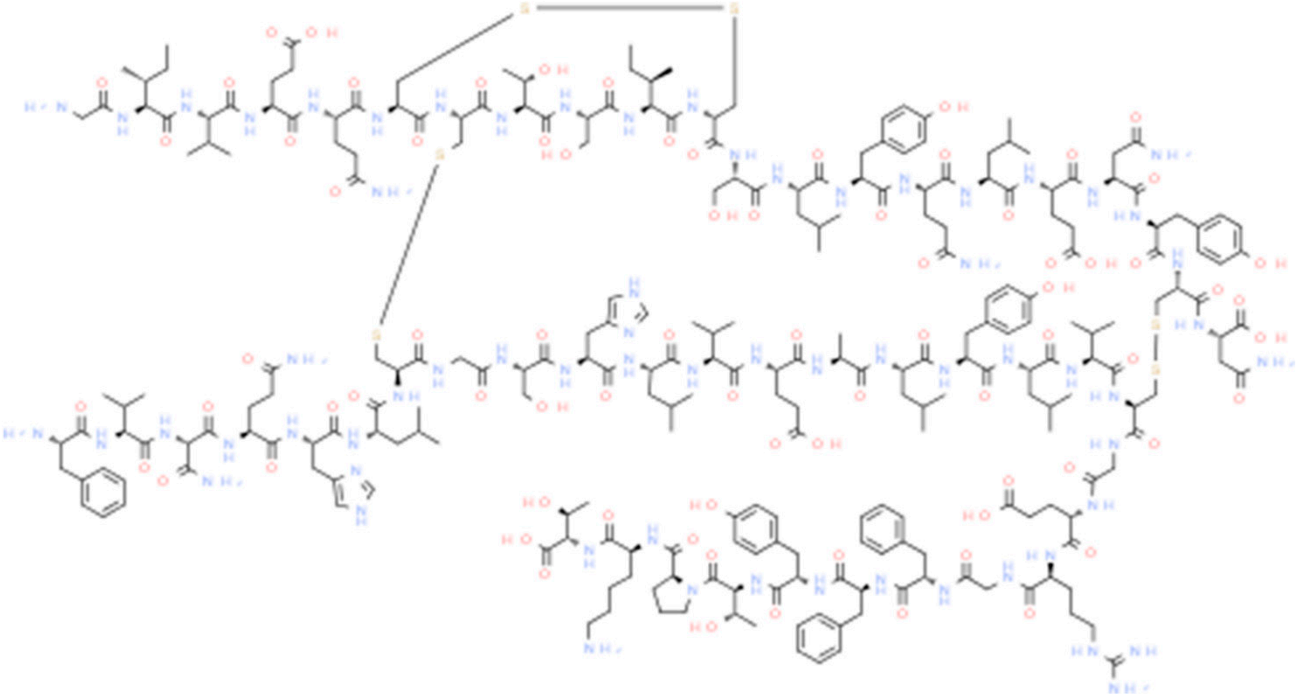

A10BA: Biguanides

A10BA02: Metformin

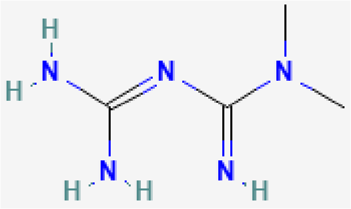

A10BG: Thiazolinidinediones

A10BG03: Pioglitazone

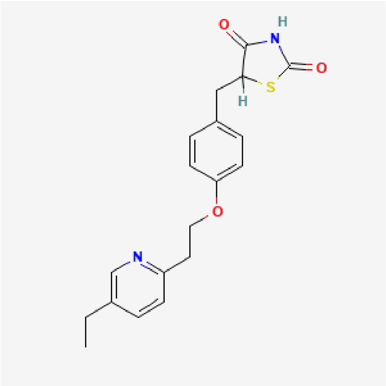

C: Cardiovascular System

C07A: Beta Blocking agents

C07AG02: Carvediol

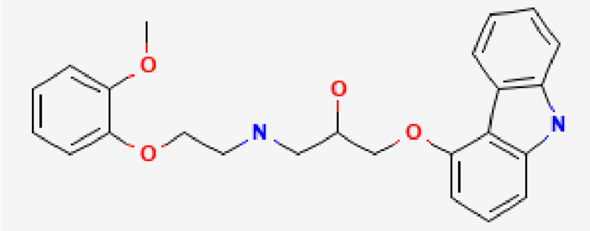

C07AB02: Metoprolol

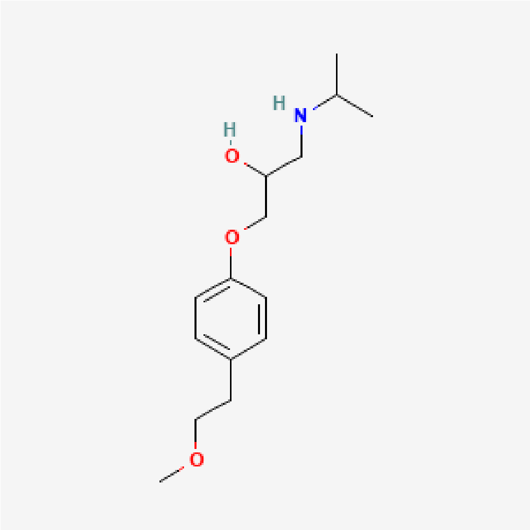

H: Systemic Hormonal Preparations

H02: Corticosteroids

Costicosterone

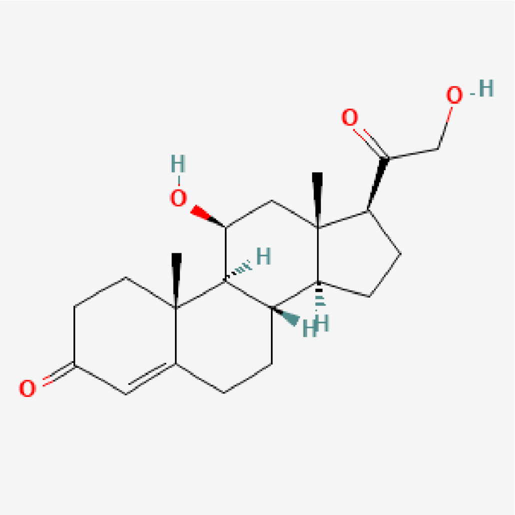

H02AB: Glucocorticoids

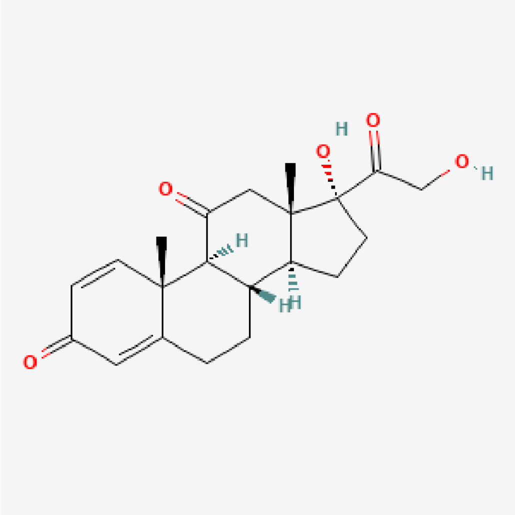

Figure S2

N: Nervous System

N02: Analgesics

N02B: Other analgesics and antipyretics

N02BF: Gabapentinoids

N02BF01: Gabapentin

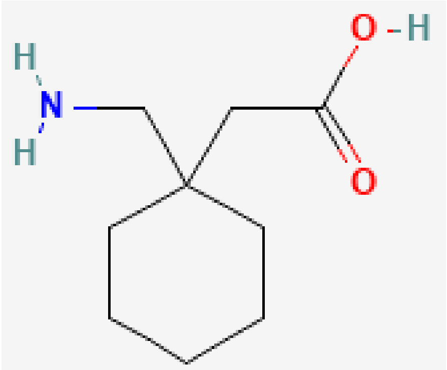

N02BF02: pregabalin

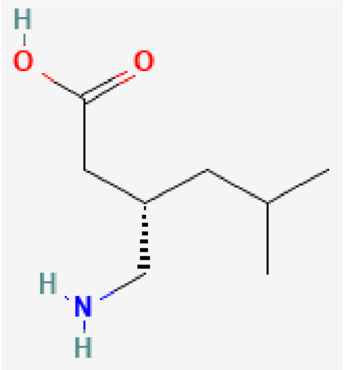

N03: Antiepileptics

N03AB: Hydantoin derivatives

N03AB02: phenytoin

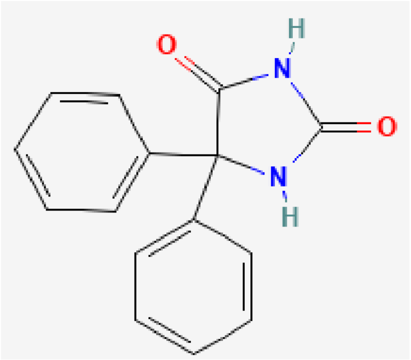

N03AF: Carboxamide derivatives
N03AF01: Carbamazepine

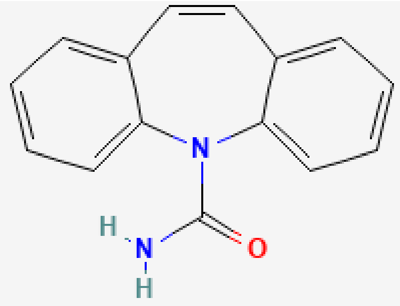

N03AG: Fatty acid derivatives
N03AG01: Valproic Acid

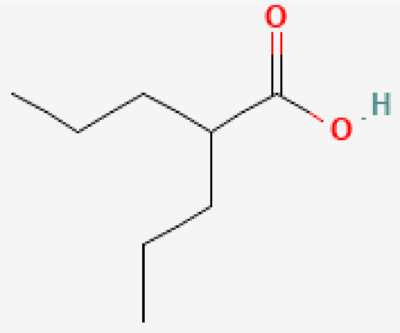

N03AX: Other antiepileptics
N03AX11: Topiramate

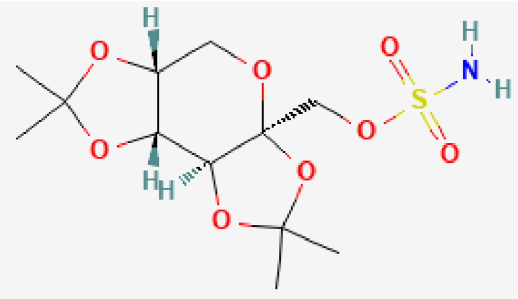

N03AX15: zonisamide

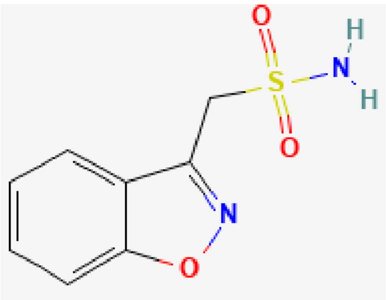

N05: Psycholeptics | N05A: Antipsychotics

N05AD: Butyrophenone derivatives
N05AD01: Haloperidol

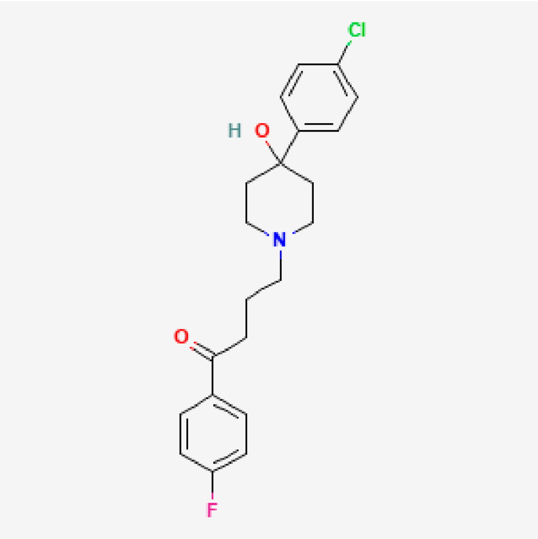

N05AE: Indole derivatives
N05AE04: Ziprasidone

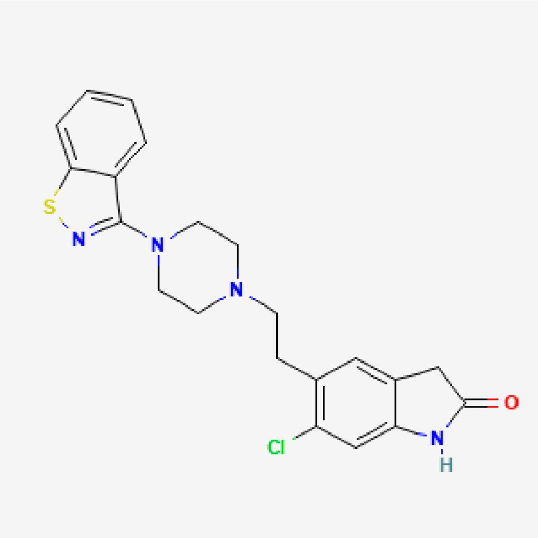

N05AL: Benzamides
N05AL05: Amisulpride

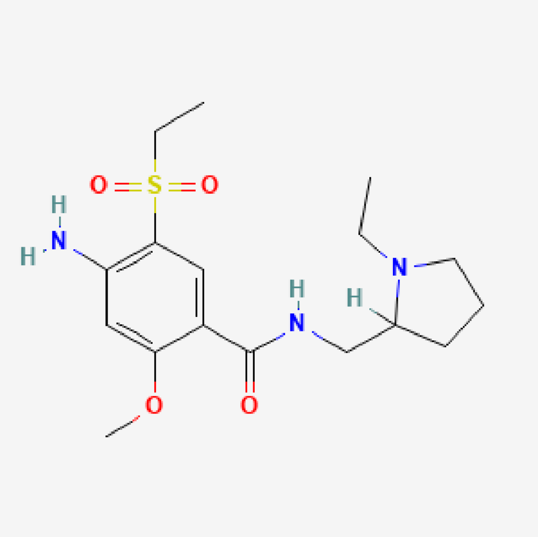

N05AX Other antipsychotics
N05AX08: Risperidone

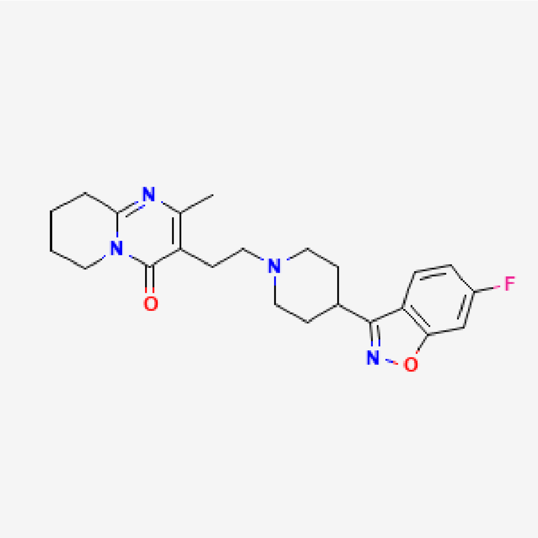

N05AX12: Aripiprazole

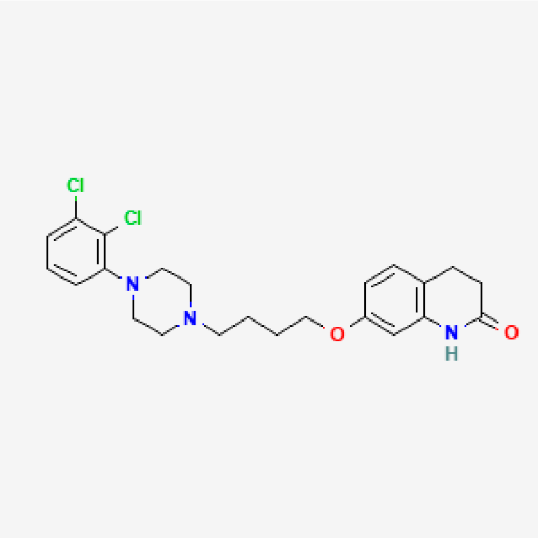

N06: Psychoanaleptics | N06A: Antidepressants

N06AA: Nonselective monoamine reuptake inhibitors

N06AA09: Amitriptyline

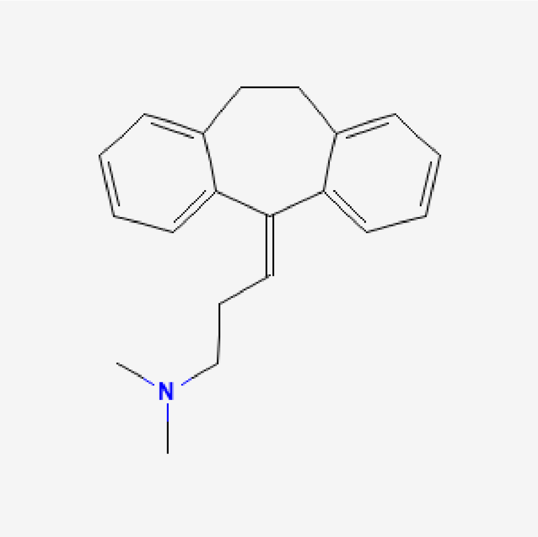

N06AA10: Nortriptyline

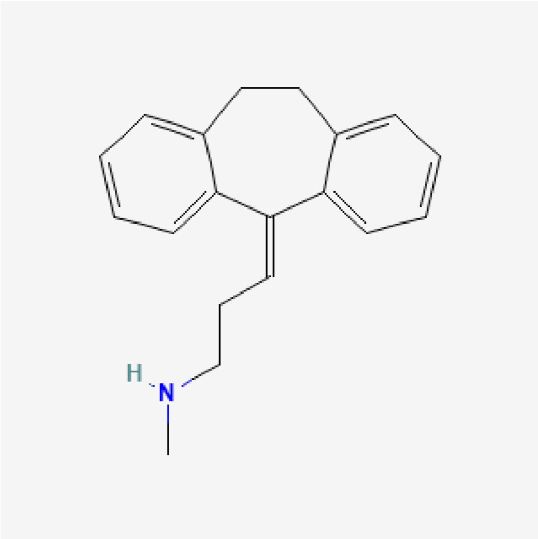

N06AB: Selective serotonina reuptake inhibitors

N06AB03: Fluoxetine

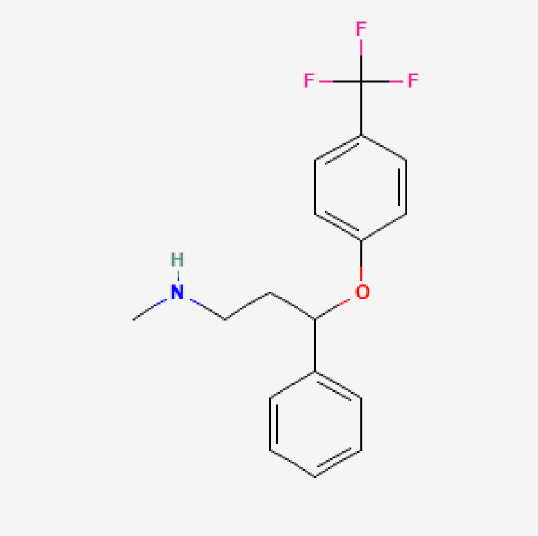

N06AB04: Citalopram

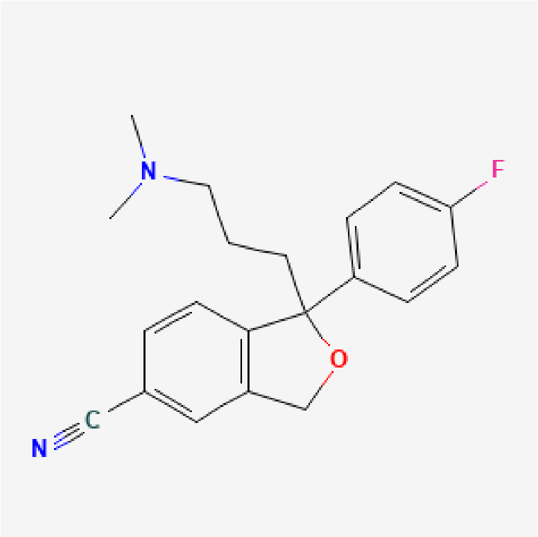

N06AB06: Sertraline

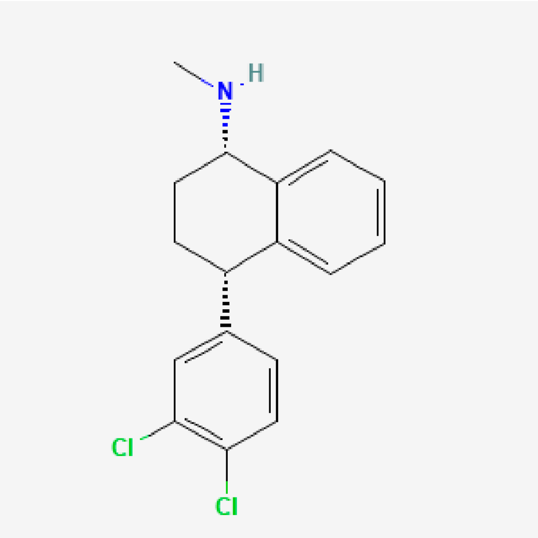

N06AX: Other antidepressants

N06AX1: Mirtazapine

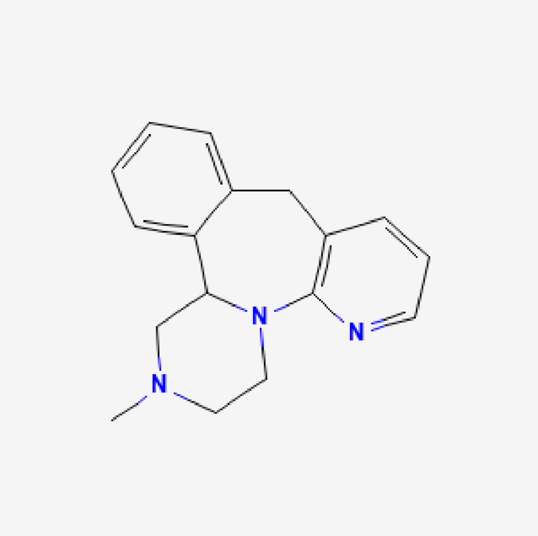

N06AX16: Venlafaxine

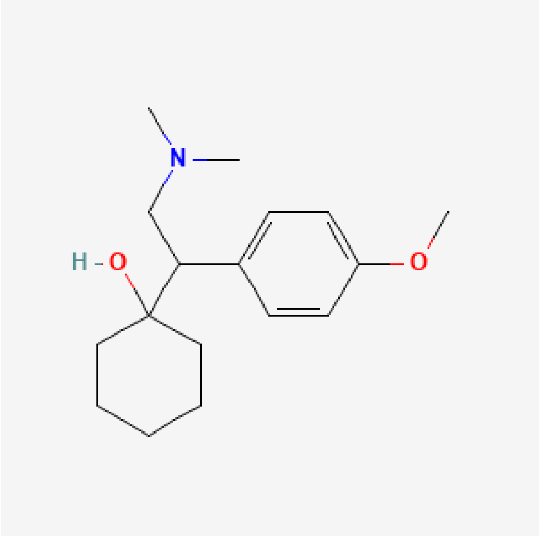

N06AX21: Duloxetine

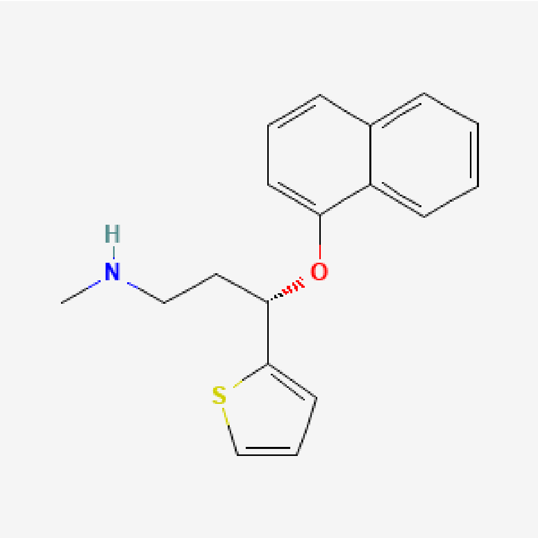

Supplement: Supplementary file 1 [file ijms-25-02324-s001.zip › ijms-2827219-supplementary.pdf]
